# 1,4-Bis(4-chloro­phen­yl)-2-hydroxy­butane-1,4-dione

**DOI:** 10.1107/S1600536810018027

**Published:** 2010-05-22

**Authors:** Yongjun Liu, Kuiwei Yang, Weiling Song, Yan Qi

**Affiliations:** a College of Chemistry and Molecular Engineering, Qingdao University of Science and Technology, Qingdao 266042, People’s Republic of China

## Abstract

In the title compound, C_16_H_12_Cl_2_O_3_, the benzene rings form a dihedral angle of 2.0 (3)°. Within the central O=C—CH_2_C(H)OH—C=O unit, the carbonyl groups are coplanar and lie to opposite sides [O—C⋯C—O = −170.1 (6)°]. In the crystal, inter­molecular O—H⋯O hydrogen bonds formed between the hydr­oxy groups lead to a supra­molecular chain along the *c* axis. In addition, the crystal packing features some very weak C—H⋯π inter­actions.

## Related literature

For the synthesis and applications of 1,4-dicarbonyl compounds, see: Ellison (1973 ▶); Hassner (1991 ▶); Ohno *et al.* (2001 ▶).
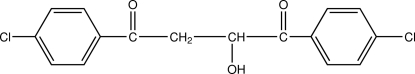


## Experimental

### 

#### Crystal data

C_16_H_12_Cl_2_O_3_
*M* *_r_* = 323.16
Monoclinic, 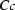

*a* = 34.800 (8) Å
*b* = 7.4221 (14) Å
*c* = 5.6535 (13) Å
β = 95.925 (2)°
*V* = 1452.4 (5) Å^3^
*Z* = 4
Mo *K*α radiation
μ = 0.45 mm^−1^
*T* = 273 K
0.12 × 0.10 × 0.08 mm

#### Data collection

Bruker SMART CCD area-detector diffractometer
Absorption correction: multi-scan (*SADABS*; Sheldrick, 1996 ▶) *T*_min_ = 0.947, *T*_max_ = 0.964
3531 measured reflections
1256 independent reflections
998 reflections with *I* > 2σ(*I*)
*R*_int_ = 0.025

#### Refinement

*R*[*F* ^2^ > 2σ(*F* ^2^)] = 0.057
*wR*(*F* ^2^) = 0.163
*S* = 1.05
1256 reflections
190 parameters
9 restraints
H-atom parameters constrained
Δρ_max_ = 0.40 e Å^−3^
Δρ_min_ = −0.21 e Å^−3^

### 

Data collection: *SMART* (Bruker, 2001 ▶); cell refinement: *SAINT* (Bruker, 2001 ▶); data reduction: *SAINT*; program(s) used to solve structure: *SHELXTL* (Sheldrick, 2008 ▶); program(s) used to refine structure: *SHELXTL*; molecular graphics: *SHELXTL*; software used to prepare material for publication: *SHELXTL*.

## Supplementary Material

Crystal structure: contains datablocks I, global. DOI: 10.1107/S1600536810018027/tk2670sup1.cif

Structure factors: contains datablocks I. DOI: 10.1107/S1600536810018027/tk2670Isup2.hkl

Additional supplementary materials:  crystallographic information; 3D view; checkCIF report

## Figures and Tables

**Table 1:** Hydrogen-bond geometry (Å, °) *Cg*1 and *Cg*2 are the centroids of the C1—C6 and C11—C16 rings, respectively.

*D*—H⋯*A*	*D*—H	H⋯*A*	*D*⋯*A*	*D*—H⋯*A*
O2—H2*A*⋯O2^i^	0.82	2.10	2.894 (6)	163
C1—H1⋯*Cg*1^ii^	0.93	2.89	3.507 (6)	125
C4—H4⋯*Cg*1^iii^	0.93	2.97	3.600 (6)	126
C13—H13⋯*Cg*2^ii^	0.93	2.88	3.517 (6)	127
C16—H16⋯*Cg*2^iii^	0.93	2.90	3.544 (6)	128

Symmetry codes: (i) 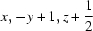; (ii) 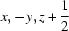; (iii) 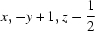.

**Fig. 1.**
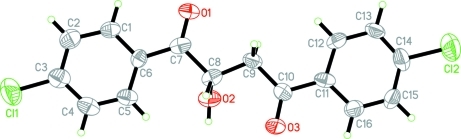
The molecular structure of the title compound, showing atom labels and 50% probability displacement ellipsoids.

**Fig. 2.**
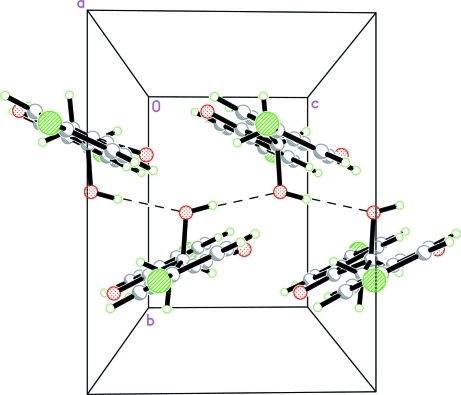
The packing diagram for the title compound, viewed down the a axis, showing the intermolecular hydrogen bonds (dashed lines).

## supplementary crystallographic information

### Comment

1,4-Dicarbonyl compounds constitute key intermediates in various natural product syntheses, and are important synthetic precursors of cyclopentenones, cyclopenta-1,3-diones, butenolides, and derivatives of furan and pyrrole (Hassner, 1991). For this reason, a number of methods for their synthesis have been developed and applied (Ellison, 1973; Ohno *et al.*, 2001).

In the title compound, Fig. 1, the benzene rings form a dihedral angle of 2.0 (3) °. Intermolecular O2—H2A···O2 hydrogen bonds lead to the formation of a supramolecular chain along the *c* axis (Table 1, Fig. 2). In addition, the crystal packing is stabilized by intermolecular C—H···π interactions (Table 1) and short Cl···Cl^i^ contacts (3.434 (3) Å for *i*: 1/2+*x*, 1/2-*y*, -1/2+*z*).

### Experimental

The title compound was obtained as a by-product in the coupling reaction between 4-ClC_6_H_4_COCH_2_Br and benzaldehyde, a reaction which is being studied in our laboratory. Colourless single crystals of the title compound suitable for X-ray diffraction were obtained by slow evaporation of an ethanol solution over a period of 20 days.

### Refinement

H atoms were positioned geometrically, with O—H = 0.82 Å and C—H = 0.95–0.99 Å, and constrained to ride on their parent atoms, with *U*_iso_(H) = 1.5 *U*_eq_(O) and *U*_iso_(H) = 1.2 *U*_eq_(C). In the absence of significant anomalous scattering effects, 1009 Friedel pairs were averaged in the final refinement.

### Crystal data

**Table:**

C_16_H_12_Cl_2_O_3_	*F*(000) = 664
*M**_r_* = 323.16	*D*_x_ = 1.478 Mg m^−^^3^
Monoclinic, *C**c*	Mo *K*α radiation, λ = 0.71073 Å
Hall symbol: C -2yc	Cell parameters from 1044 reflections
*a* = 34.800 (8) Å	θ = 2.4–24.4°
*b* = 7.4221 (14) Å	µ = 0.45 mm^−^^1^
*c* = 5.6535 (13) Å	*T* = 273 K
β = 95.925 (2)°	Column, colourless
*V* = 1452.4 (5) Å^3^	0.12 × 0.10 × 0.08 mm
*Z* = 4	

### Data collection

**Table:**

Bruker SMART CCD area-detector diffractometer	1256 independent reflections
Radiation source: fine-focus sealed tube	998 reflections with *I* > 2σ(*I*)
graphite	*R*_int_ = 0.025
phi and ω scans	θ_max_ = 25.0°, θ_min_ = 2.4°
Absorption correction: multi-scan (*SADABS*; Sheldrick, 1996)	*h* = −38→40
*T*_min_ = 0.947, *T*_max_ = 0.964	*k* = −7→8
3531 measured reflections	*l* = −6→6

### Refinement

**Table:**

Refinement on *F*^2^	Secondary atom site location: difference Fourier map
Least-squares matrix: full	Hydrogen site location: inferred from neighbouring sites
*R*[*F*^2^ > 2σ(*F*^2^)] = 0.057	H-atom parameters constrained
*wR*(*F*^2^) = 0.163	*w* = 1/[σ^2^(*F*_o_^2^) + (0.1063*P*)^2^ + 0.4654*P*] where *P* = (*F*_o_^2^ + 2*F*_c_^2^)/3
*S* = 1.05	(Δ/σ)_max_ < 0.001
1256 reflections	Δρ_max_ = 0.40 e Å^−^^3^
190 parameters	Δρ_min_ = −0.21 e Å^−^^3^
9 restraints	Absolute structure: nd
Primary atom site location: structure-invariant direct methods	

### Special details

**Table:**

Geometry. All esds (except the esd in the dihedral angle between two l.s. planes) are estimated using the full covariance matrix. The cell esds are taken into account individually in the estimation of esds in distances, angles and torsion angles; correlations between esds in cell parameters are only used when they are defined by crystal symmetry. An approximate (isotropic) treatment of cell esds is used for estimating esds involving l.s. planes.
Refinement. Refinement of *F*^2^ against ALL reflections. The weighted *R*-factor wR and goodness of fit *S* are based on *F*^2^, conventional *R*-factors *R* are based on *F*, with *F* set to zero for negative *F*^2^. The threshold expression of *F*^2^ > σ(*F*^2^) is used only for calculating *R*-factors(gt) etc. and is not relevant to the choice of reflections for refinement. *R*-factors based on *F*^2^ are statistically about twice as large as those based on *F*, and *R*- factors based on ALL data will be even larger.

### Fractional atomic coordinates and isotropic or equivalent isotropic displacement parameters (Å^2^)

**Table:**

	*x*	*y*	*z*	*U*_iso_*/*U*_eq_	
C1	1.04214 (18)	0.1713 (8)	0.3400 (11)	0.0369 (15)	
H1	1.0356	0.1204	0.4809	0.044*	
C2	1.0810 (2)	0.1845 (10)	0.2991 (14)	0.053 (2)	
H2	1.1002	0.1385	0.4089	0.063*	
C3	1.0905 (2)	0.2672 (9)	0.0921 (14)	0.049 (2)	
C4	1.0605 (2)	0.3249 (10)	−0.0726 (13)	0.049 (2)	
H4	1.0664	0.3745	−0.2154	0.058*	
C5	1.0234 (2)	0.3116 (8)	−0.0327 (12)	0.0403 (16)	
H5	1.0043	0.3556	−0.1448	0.048*	
C6	1.0133 (2)	0.2332 (8)	0.1732 (12)	0.0357 (16)	
C7	0.9724 (2)	0.2035 (8)	0.2253 (12)	0.0374 (16)	
C8	0.9403 (2)	0.2685 (8)	0.0412 (13)	0.0383 (14)	
H8	0.9443	0.2140	−0.1121	0.046*	
C9	0.9007 (2)	0.2137 (9)	0.1027 (12)	0.0412 (15)	
H9A	0.8999	0.0839	0.1210	0.049*	
H9B	0.8961	0.2678	0.2533	0.049*	
C10	0.8693 (2)	0.2712 (9)	−0.0860 (13)	0.0454 (18)	
C11	0.8287 (2)	0.2621 (8)	−0.0279 (12)	0.0362 (16)	
C12	0.8185 (2)	0.1854 (9)	0.1838 (11)	0.0444 (18)	
H12	0.8377	0.1432	0.2964	0.053*	
C13	0.7794 (2)	0.1712 (10)	0.2287 (12)	0.0465 (19)	
H13	0.7725	0.1146	0.3649	0.056*	
C14	0.7524 (2)	0.2427 (10)	0.0672 (14)	0.053 (2)	
C15	0.7606 (2)	0.3157 (9)	−0.1456 (15)	0.0489 (18)	
H15	0.7409	0.3558	−0.2564	0.059*	
C16	0.7982 (2)	0.3280 (9)	−0.1908 (12)	0.0473 (18)	
H16	0.8040	0.3809	−0.3319	0.057*	
Cl1	1.13745 (6)	0.2822 (4)	0.0298 (3)	0.0922 (9)	
Cl2	0.70386 (6)	0.2245 (4)	0.1208 (4)	0.0948 (10)	
O1	0.96490 (15)	0.1328 (6)	0.4095 (9)	0.0515 (12)	
O2	0.94303 (14)	0.4587 (5)	0.0178 (8)	0.0501 (10)	
H2A	0.9434	0.5058	0.1492	0.075*	
O3	0.87583 (16)	0.3179 (10)	−0.2824 (10)	0.084 (2)	

### Atomic displacement parameters (Å^2^)

**Table:**

	*U*^11^	*U*^22^	*U*^33^	*U*^12^	*U*^13^	*U*^23^
C1	0.043 (4)	0.037 (3)	0.030 (3)	−0.007 (3)	0.002 (3)	−0.001 (3)
C2	0.066 (5)	0.056 (5)	0.035 (4)	0.005 (4)	0.000 (4)	0.001 (3)
C3	0.060 (6)	0.047 (4)	0.040 (4)	−0.008 (3)	0.000 (4)	−0.012 (3)
C4	0.057 (6)	0.053 (4)	0.037 (4)	−0.006 (3)	0.008 (4)	−0.001 (3)
C5	0.047 (4)	0.033 (3)	0.040 (4)	−0.009 (3)	0.001 (3)	0.002 (3)
C6	0.042 (4)	0.033 (3)	0.033 (4)	0.009 (3)	0.008 (3)	−0.001 (3)
C7	0.057 (4)	0.026 (3)	0.030 (4)	0.006 (3)	0.009 (3)	−0.001 (3)
C8	0.048 (3)	0.033 (3)	0.033 (3)	0.011 (2)	0.000 (3)	0.002 (3)
C9	0.047 (3)	0.047 (4)	0.031 (3)	−0.007 (3)	0.008 (3)	0.003 (3)
C10	0.043 (4)	0.057 (4)	0.037 (4)	0.006 (3)	0.008 (3)	0.003 (3)
C11	0.047 (4)	0.029 (3)	0.032 (4)	−0.010 (2)	0.003 (3)	−0.004 (3)
C12	0.053 (5)	0.055 (4)	0.026 (3)	0.013 (3)	0.004 (3)	0.006 (3)
C13	0.054 (5)	0.058 (4)	0.029 (3)	−0.017 (4)	0.013 (3)	0.005 (3)
C14	0.034 (4)	0.069 (5)	0.058 (6)	−0.011 (3)	0.014 (4)	−0.004 (4)
C15	0.027 (3)	0.055 (4)	0.062 (5)	−0.008 (3)	−0.011 (3)	0.003 (4)
C16	0.061 (5)	0.042 (3)	0.037 (4)	0.001 (3)	−0.002 (4)	0.004 (3)
Cl1	0.0472 (14)	0.147 (3)	0.0842 (18)	−0.0177 (14)	0.0152 (13)	0.0093 (17)
Cl2	0.0469 (15)	0.150 (3)	0.089 (2)	−0.0122 (14)	0.0178 (13)	0.0072 (17)
O1	0.052 (3)	0.057 (3)	0.047 (3)	0.002 (2)	0.012 (2)	0.019 (2)
O2	0.064 (3)	0.039 (2)	0.048 (2)	0.0037 (19)	0.0072 (18)	0.0049 (19)
O3	0.048 (3)	0.172 (6)	0.033 (3)	0.010 (3)	0.011 (2)	0.033 (3)

### Geometric parameters (Å, °)

**Table:**

C1—C6	1.384 (10)	C9—C10	1.507 (11)
C1—C2	1.398 (10)	C9—H9A	0.9700
C1—H1	0.9300	C9—H9B	0.9700
C2—C3	1.391 (12)	C10—O3	1.206 (9)
C2—H2	0.9300	C10—C11	1.485 (10)
C3—C4	1.393 (11)	C11—C12	1.404 (9)
C3—Cl1	1.711 (8)	C11—C16	1.419 (10)
C4—C5	1.334 (9)	C12—C13	1.413 (9)
C4—H4	0.9300	C12—H12	0.9300
C5—C6	1.380 (9)	C13—C14	1.349 (11)
C5—H5	0.9300	C13—H13	0.9300
C6—C7	1.499 (10)	C14—C15	1.376 (12)
C7—O1	1.218 (8)	C14—Cl2	1.753 (8)
C7—C8	1.525 (10)	C15—C16	1.361 (10)
C8—O2	1.422 (7)	C15—H15	0.9300
C8—C9	1.509 (7)	C16—H16	0.9300
C8—H8	0.9800	O2—H2A	0.8200
			
C6—C1—C2	120.7 (6)	C10—C9—H9A	109.3
C6—C1—H1	119.7	C8—C9—H9A	109.3
C2—C1—H1	119.7	C10—C9—H9B	109.3
C3—C2—C1	119.2 (7)	C8—C9—H9B	109.3
C3—C2—H2	120.4	H9A—C9—H9B	107.9
C1—C2—H2	120.4	O3—C10—C11	119.3 (6)
C2—C3—C4	118.1 (8)	O3—C10—C9	122.8 (6)
C2—C3—Cl1	121.1 (6)	C11—C10—C9	117.8 (6)
C4—C3—Cl1	120.6 (6)	C12—C11—C16	117.0 (7)
C5—C4—C3	122.3 (7)	C12—C11—C10	122.6 (6)
C5—C4—H4	118.8	C16—C11—C10	120.4 (6)
C3—C4—H4	118.8	C11—C12—C13	121.2 (6)
C4—C5—C6	120.7 (7)	C11—C12—H12	119.4
C4—C5—H5	119.7	C13—C12—H12	119.4
C6—C5—H5	119.7	C14—C13—C12	117.7 (6)
C5—C6—C1	118.9 (7)	C14—C13—H13	121.2
C5—C6—C7	124.0 (6)	C12—C13—H13	121.2
C1—C6—C7	117.1 (6)	C13—C14—C15	123.6 (7)
O1—C7—C6	121.4 (6)	C13—C14—Cl2	117.9 (6)
O1—C7—C8	120.9 (7)	C15—C14—Cl2	118.2 (6)
C6—C7—C8	117.6 (6)	C16—C15—C14	118.8 (7)
O2—C8—C9	111.2 (5)	C16—C15—H15	120.6
O2—C8—C7	109.0 (5)	C14—C15—H15	120.6
C9—C8—C7	112.3 (5)	C15—C16—C11	121.6 (7)
O2—C8—H8	108.1	C15—C16—H16	119.2
C9—C8—H8	108.1	C11—C16—H16	119.2
C7—C8—H8	108.1	C8—O2—H2A	109.5
C10—C9—C8	111.8 (5)		

### Hydrogen-bond geometry (Å, °)

**Table:**

Cg1 and Cg2 are the centroids of the C1—C6 and C11—C16 rings, respectively.

**Table:**

*D*—H···*A*	*D*—H	H···*A*	*D*···*A*	*D*—H···*A*
O2—H2A···O2^i^	0.82	2.10	2.894 (6)	163
C1—H1···Cg1^ii^	0.93	2.89	3.507 (6)	125
C4—H4···Cg1^iii^	0.93	2.97	3.600 (6)	126
C13—H13···Cg2^ii^	0.93	2.88	3.517 (6)	127
C16—H16···Cg2^iii^	0.93	2.90	3.544 (6)	128

Symmetry codes: (i) *x*, −*y*+1, *z*+1/2; (ii) *x*, −*y*, *z*+1/2; (iii) *x*, −*y*+1, *z*−1/2.
